# Wideband helicity dependent spoof surface plasmon polaritons coupling metasurface based on dispersion design

**DOI:** 10.1038/srep38460

**Published:** 2016-12-06

**Authors:** Guoxiang Dong, Hongyu Shi, Yuchen He, Anxue Zhang, Xiaoyong Wei, Yongyong Zhuang, Bai Du, Song Xia, Zhuo Xu

**Affiliations:** 1 Electronic Materials Research Laboratory, Key Laboratory of the Ministry of Education, Xi’an Jiaotong University, Xi’an 710049, China; 2 School of Electronic and Information Engineering, Xi’an Jiaotong University, Xi’an, 710049, China

## Abstract

The surface plasmon polaritons (SPPs) have many potential application due to their local field enhancement and sub-wavelength characteristics. Recently, the gradient metasurface is introduced to couple the spoof SPPs in microwave frequency band. One of the most important issue which should be solved is the narrowband of spoof SPPs coupling on the gradient metasurface. Here, the metasurface is proposed to achieve the wideband helicity dependent directional spoof SPPs coupling for circular polarized light. Our research show that the coupling frequency of spoof SPPs on the gradient metasurface is determined by the dispersion of the metasurface, so the coupling frequency can be controlled by dispersion design. The careful design of each cell geometric parameters has provided many appropriate dispersion relations possessed by just one metasurface. The wave vector matching between the propagating wave and the spoof SPPs has been achieved at several frequencies for certain wave vector provided by the metasurface, which leads to wideband spoof SPPs coupling. This work has shown that wideband helicity dependent directional spoof SPPs coupling has been achieved with a high efficiency. Hence, the proposed wideband spoof SPPs coupling presents the improvement in practice applications.

The surface plasmon polaritons (SPPs) are special electromagnetic waves at metal/dielectric or metal/air interface^1^, which are bounded by and propagate along the interface, and also possess a lager wave vector than the wave vector of the free space wave. Natural SPPs can be excited in the optical frequency band, where the metal behaves like plasma with negative permittivity. The SPPs have many significant practical applications because of their highly localized field enhancement and excellent sub-wavelength confinement such as usage in highly integrated optical circuits, high-resolution imaging, and wave plates. When the frequency is reduced to terahertz and microwave frequency range, natural SPPs do not exist. However, researchers have done great efforts to excite the SPPs in these frequency ranges. The plasmonic metamaterials^2^,^3^,^4^,^5^,^6^,^7^, including periodic grooves^7^, holes^8^,^9^, and corrugated metallic stripes, have been designed and proven to support and propagate the SPPs-like mode, which are known as the spoof SPPs. Such spoof SPPs can be used in high directivity antennas, cloaks, and miniature microwave devices.

In order to excite SPPs, bulky prisms^10^,^11^ or dielectric gratings^12^ which suffer from very inefficiency are usually used in optical frequency. Moreover, the generation of the SPPs requires the correct angle of incidence in order to meet the required wave vector of the SPPs. Recently, metasurfaces^13^,^14^,^15^,^16^,^17^,^18^,^19^,^20^,^21^,^22^ which present two dimensional metamaterials have been proposed to control electromagnetic (EM) waves. Moreover, the anomalous reflection or refractions^23^,^24^,^25^,^26^, the polarization conversion^18^, and the focusing can be realized by the metasurface. Particularly, the generalized Snell’s law predicts that the spoof SPPs can be excited on the gradient metasurface, when the pre-defined wave vector of the gradient metasurface is greater than the wave vector of free space wave, even for normal angle of incidence^15^. Some significant works have been done for exciting spoof SPPs based on the gradient metasurface^27^, such as polarization-independent coupling using periodic array^28^,^29^ and high-efficiency spoof SPPs coupling for linear polarized waves^15^,^30^. However, all of the previous works on the spoof SPPs coupling work at a single frequency, which greatly limits their practical application.

In this work, 1 GHz bandwidth spoof SPPs coupling from 6.6 GHz to 7.6 GHz based on the gradient metasurface with dispersion design is presented. The previous works^15^,^28^,^29^,^30^ related to the spoof SPPs excitation on the gradient metasurface are focused on the metasurface gradient phase design, while our work studies the dispersion relation for the spoof SPPs excitation on the gradient metasurface, and it has been shown that the spoof SPPs coupling frequency point is determined by the dispersion relation of the metasurface under given wave vector. Due to that, the coupling frequency can be artificially controlled by the metasurface dispersion design. In our work, the metasurface has the gradient phase distribution according to the Pancharatnam-Berry phase^31^. However, the carefully optimized geometrical parameters of the cells in our design, which are identical to the Pancharatnam-Berry metasurface, vary from one to another, and because of that the metasurface has several appropriate dispersion properties suitable to excite the spoof SPPs. Thus, the spoof SPPs coupling at multiple frequencies is achieved, which leads to 1 GHz bandwidth spoof SPPs coupling from 6.6 GHz to 7.6 GHz for circular polarization wave. The wide band helicity dependent directional spoof SPPs coupling with a high efficiency is achieved in our work.

## Theoretical background

**Figure 1:**
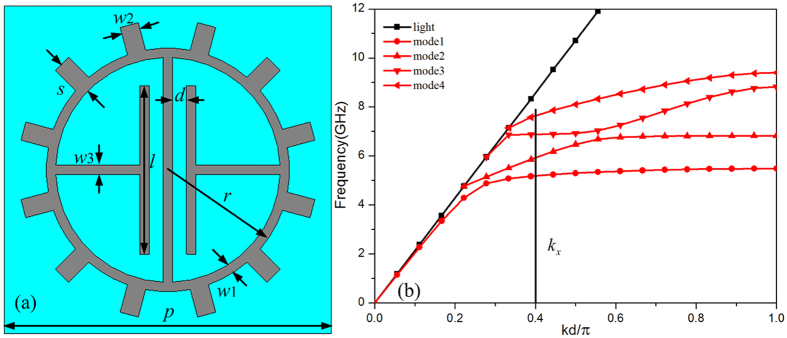
(**a**) Proposed structure of the cell. (**b**) Dispersion curves for the cell.

The cell of the proposed metasurface is essentially a sub-wavelength metallic structure on a grounded substrate as shown in Fig. 1(a). The permittivity of dielectric is *ε* = 6.15 + i0.0038, and the thickness of the substrate *h* is equal to 3.18 mm. The geometrical parameters of the metasurface cell are: *p* = 7 mm, *r* = 2.6 mm, *l* = 3.6 mm, *w1* = 0.2 mm, *w2* = 0.4 mm, *w3* = 0.2 mm and *d* = 0.3 mm. In order to research the excitation of the spoof SPPs, the dispersion property of the spoof SPP on the proposed metasurface cell is investigated^32^ in Fig. 1(b). Four dispersion curves exist, and a difference between the dispersion curves and the light line exists at a given frequency, which indicates that the EM waves can be confined on the metasurface^4^,^5^,^6^. The lager difference indicates stronger confinement of the spoof SPPs on the metasurface. Thus, the proposed metasurface can support and propagate the spoof SPPs with different modes. The spoof SPPs supported by the metasurface has a lager wave vector *k*_*x*_ than the free space wave at a given frequency, which indicates that the wave vector of the incident free space wave should be increased to couple the incident free space wave into spoof SPPs on the metasurface. In our work, the proposed metasurface is a gradient metasurface, which is able to provide an additional wave vector^15^,^30^ to compensate the difference of the wave vectors between the incident free space wave and the spoof SPPs on the metasurface for exciting the spoof SPPs on the metasurface.

The wave vector of the coupled spoof SPPs on the gradient metasurface is entirely provided by the metasurface^15^,^30^ for normal incidence. In order to excite these spoof SPPs supported by the proposed metasurface for normal incidence, the metasurface should entirely provide the wave vector *k*_*x*_, which is required by these spoof SPPs shown in Fig. 1(b). Notably, the wave vector of these spoof SPPs *k*_*x*_ is larger than the wave vector of the free space wave, so the metasurface should provide a larger wave vector than free space wave. This is consistent well with the generalized Snell’s law. The generalized Snell’s law indicates that the incident free space wave with normal incident angle^15^ can convert into the spoof SPPs, when the wave vector provided by the gradient metasurface is greater than the wave vector of incident free space wave. These spoof SPPs supported by the proposed metasurface has defined dispersion relations shown in Fig. 1(b). Although the metasurface can provide larger wave vector *k*_*x*_ required by these spoof SPPs from 5 GHz to 8 Ghz, the spoof SPPs coupling occurs only at frequencies, which correspond to the intersections of the dispersion curves of the spoof SPPs and the line of the wave vector *k*_*x*_. Thus, the coupling frequency of the spoof SPPs on the gradient metasurface can be ascertained by the dispersion relation for a given wave vector *k*_*x*_. In a word, the metasurface can entirely provide the required wave vector of the coupled spoof SPPs, and the coupled spoof SPPs propagating on the metasurface have a defined dispersion^32^.

For a given wave vector *k*_*x*_ in the wideband frequency range (5 GHz to 8 GHz) as shown in Fig. 1(b), the spoof SPPs coupling just occurs at corresponding frequency of the intersections of the dispersion curves of the spoof SPPs and the line of the wave vector *k*_*x*_, which indicates that we can control the coupling frequency by dispersion design. If the metasurface possesses multiple appropriate dispersion relations, wideband spoof SPPs coupling is able to realize in a frequency band formed by the nearby frequencies, at which the spoof SPPs coupling occurs. Thus, wideband spoof SPPs coupling can be excited on the gradient metasurface, which can provide a larger wave vector in a wide frequency band and have multiple appropriate dispersion relations in this band range, simultaneity. In our work, the proposed metasurface is gradient metasurface which can provide a lager wave vector. The phase gradient of the proposed metasurface is achieved by cells rotation, which is like the Pancharatnam-Berry phase metasurface. However, the geometrical parameters of each cell, which have identical size for the Pancharatnam-Berry metasurface, are carefully designed different with each other. This is due to the fact that the dispersion properties are related to the geometrical parameters of cell.

## Metasurface design and simulation

**Figure 2:**
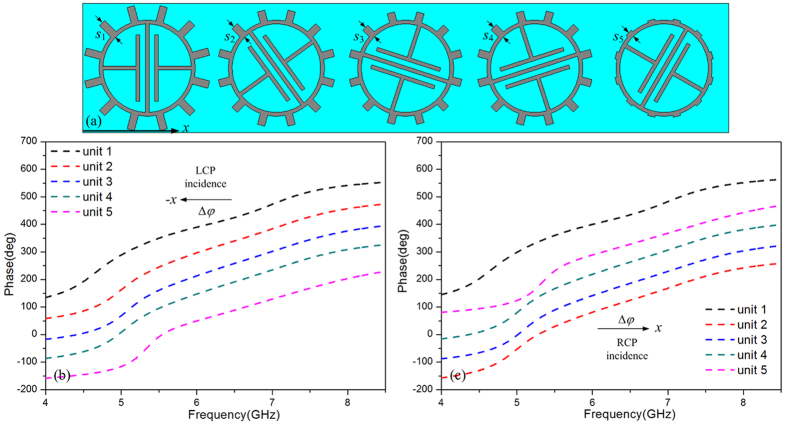
(**a**) The proposed metasurface, (**b**) Reflection phase distributions for left circularly polarized wave illumination, (**c**) Reflection phase distributions for right circularly polarized wave illumination.

The super unit cell of the proposed metasurface includes five cells, and each cell have a different orientation angle as shown in Fig. 2(a). The proposed metasurface is similar to the Pancharatnam-Berry phase metasurface, but the length of the proposed metasurface cell, *s*, is designed in the way that different cells have different length in order to achieve multiple appropriate dispersion relations. The length *s* of each cell is 1.05 mm, 0.8 mm, 0.7 mm, 0.75 mm and 0.3 mm, respectively. The phase change of a super cell is 2*π* along the *x* direction, then the phase gradient of the metasurface is |*ξ*| = |*dϕ/dx*| = 2*π*/5*p*. According to the generalized law of reflection, the gradient metasurface provide an additional wave vector in x-direction^15^,^24^,^30^, that is


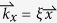


As it is well known, the gradient phase of the Pancharatnam-Berry metasurface is achieved by rotating cells in a constant angle step. However, in order to generate the sufficient phase distribution within the wideband, the orientation angles along *x* direction of the proposed metasurface cells are 0, 36, 72, 108 and 150 degrees, respectively. Figure 2(b) and (c) show the reflection phases for each cell, which confirm that the phase gradient can be achieved within the wideband (5.5 GHz to 8 GHz). The direction of phase gradient is controlled by the helicity of the incidence^31^,^32^, the phase gradient Δ*φ* is along -*x* direction for left circularly polarized wave, while the phase gradient Δ*φ* is along *x* direction for right circularly polarized wave. Thus, the wave vector 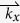 provided by the proposed metasurface have opposite direction for different helicity incidence.

**Figure 3:**
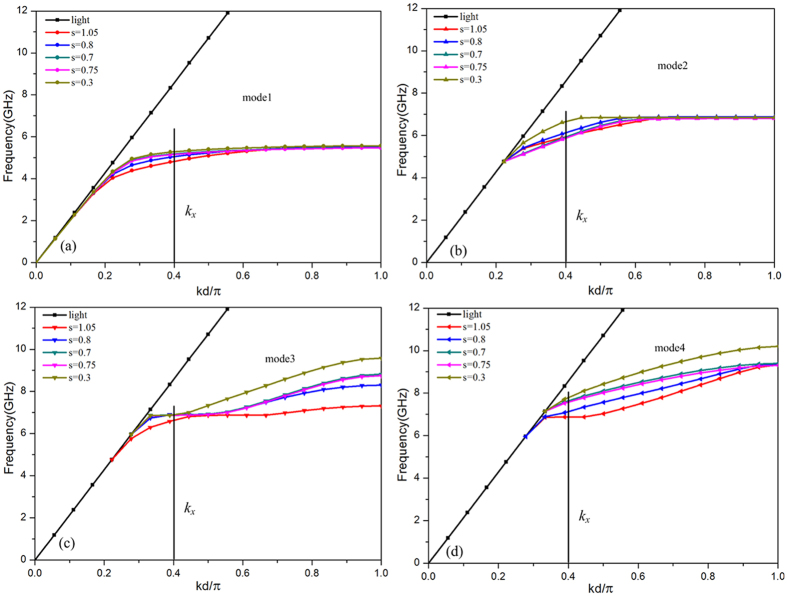
The dispersion relations of the spoof SPP on the cell with different lengths *s*, and in vacuum. (**a**) Dispersion relation of the mode 1. (**b**) Dispersion relation of the mode 2. (**c**) Dispersion relation of the mode 3 (**d**) Dispersion relation of the mode 4.

Figure 3 present the four modes of the spoof SPPs supported by the proposed metasurface, it is clearly shown that the length *s* has an effect on dispersion relation of each mode. Multiple different dispersion relations are obtained by changing the length *s*, thus, different coupling frequencies of the spoof SPPs can be achieved under a given wave vector *k*_*x*_. The cells with different length *s* comprise the proposed metasurface shown in Fig. 2(a), so the proposed metasurface possesses these multiple different dispersion relations simultaneously. The metasurface can provide a wave vector from 5.5 GHz to 8 GHz as presented in Fig. 2(b) and (c). Thus, we can predict that the spoof SPPs coupling occurs at multiple frequencies, which can lead to wideband spoof SPPs coupling.

**Figure 4:**
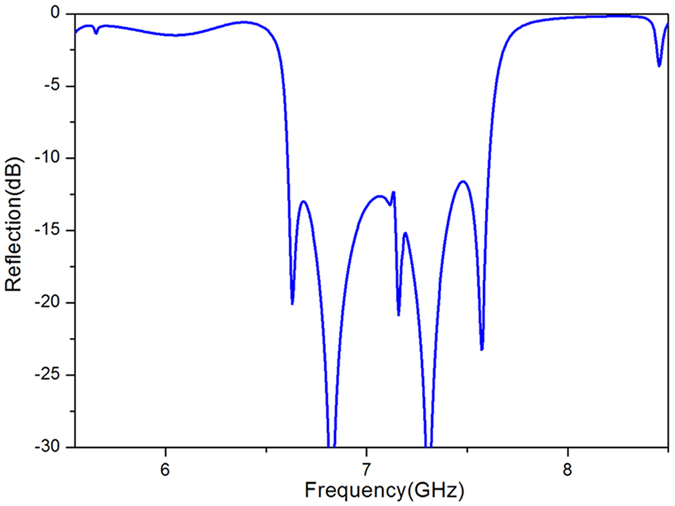
Simulated reflection of the proposed metasurface.

The performances of the spoof SPPs coupling on the proposed metasurface are numerically investigated by frequency-domain analysis obtained by the commercially available software CST MICROWAVE STUDIO. For the simulation, the four boundaries in *x* and *y* directions are set as periodical boundaries, and the absorbing boundary condition was used for the *z* direction. Figure 4 shows the simulated reflection versus frequency of the metasurface under both right circularly polarized wave and left circularly polarized wave. The simulation results indicate that the reflection is reduced by more than 10 dB from 6.6 GHz to 7.6 GHz. This 10 dB bandwidths contains four dips at 6.64 GHz, 6.835 GHz, 7.33 GHz and 7.574 GHz. These dips are results of the spoof SPPs coupling. The frequency located at the dip is the coupling frequency of the spoof SPPs and it has corresponding dispersion curves shown in Fig. 3. The dip at 6.64 GHz correspond to the spoof SPP coupling in mode 2, and the corresponding dispersion curves is *s* = 0.3, Fig. 3(b). The dip at 6.835 GHz is also result of the spoof SPPs coupling in mode 3, while the corresponding dispersion curves are *s* = 0.8, *s* = 0.7, *s* = 0.75 and *s* = 0.3, Fig. 3(c). The spoof coupling at 7.33 GHz is in mode 4, and the corresponding dispersion curves is *s* = 0.8, Fig. 3(d). The spoof coupling at 7.574 GHz is also in mode 4, while the corresponding dispersion curve is *s* = 0.7 and *s* = 0.75 Fig. 3(d). The 10 dB bandwidth is formed by spoof SPPs coupling at nearby frequencies, these nearby coupling frequencies benefit from the dispersion design of the proposed metasurface. Moreover, the reflection is reduced by more than 20 dB at 6.64 GHz, 6.835 GHz, 7.33 GHz and 7.574 GHz, which corresponds to a high efficiency.

**Figure 5:**
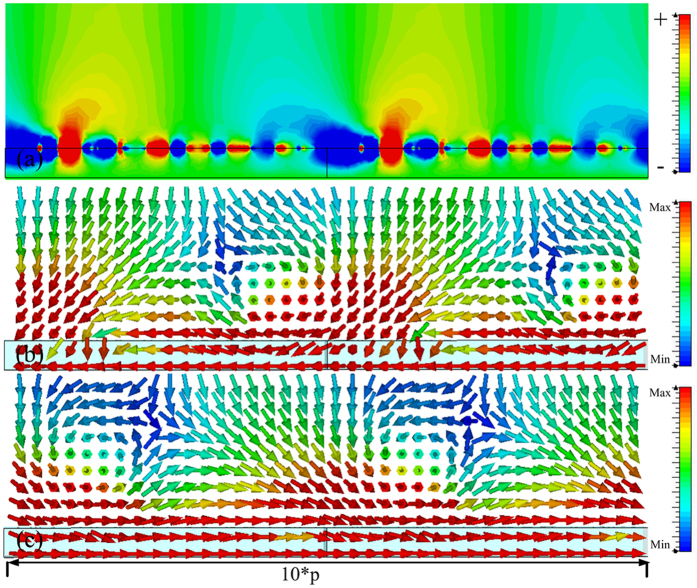
(**a**) The magnetic field in *z* direction of the spoof SPP at 6.64 GHz. (**b**) The power flow for left circularly polarized wave illumination at 6.64 GHz. (**c**) The power flow for right circularly polarized wave illumination at 6.64 GHz.

**Figure 6:**
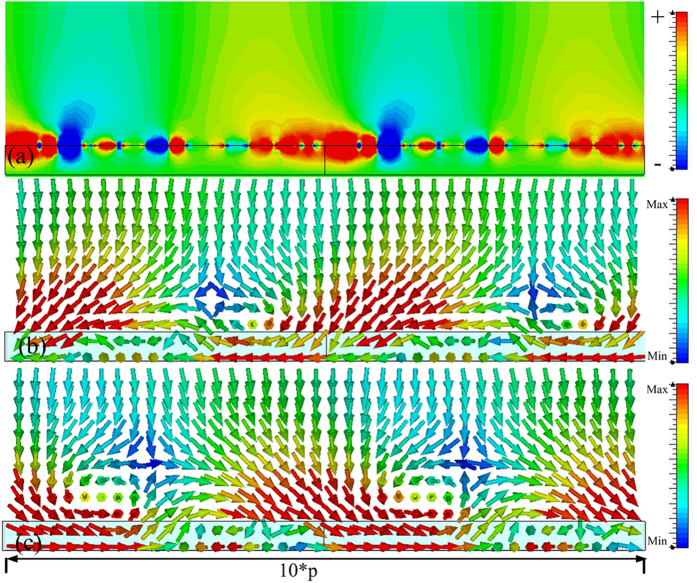
(**a**) The magnetic field in *z* direction of the spoof SPP at 6.835 GHz. (**b**) The power flow for left circularly polarized wave illumination at 6.835 GHz. (**c**) The power flow for right circularly polarized wave illumination at 6.835 GHz.

**Figure 7:**
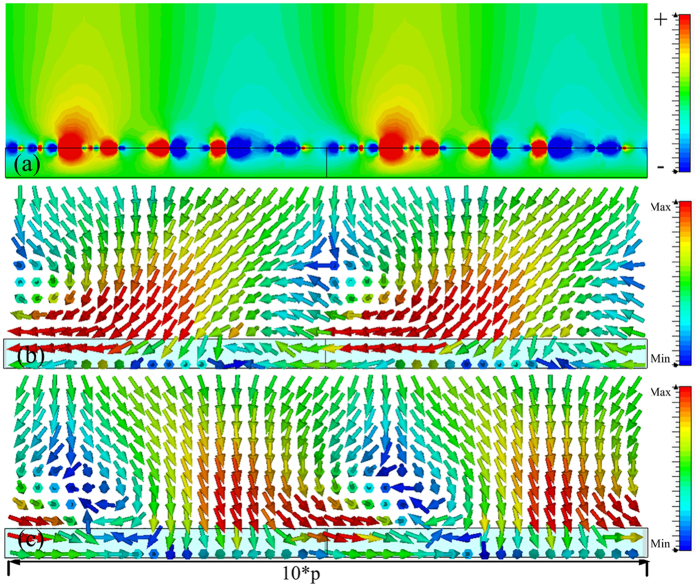
(**a**) The magnetic field in *z* direction of the spoof SPP at 7.33 GHz. (**b**) The power flow for left circularly polarized wave illumination at 7.33 GHz. (**c**) The power flow for right circularly polarized wave illumination at 7.33 GHz.

**Figure 8:**
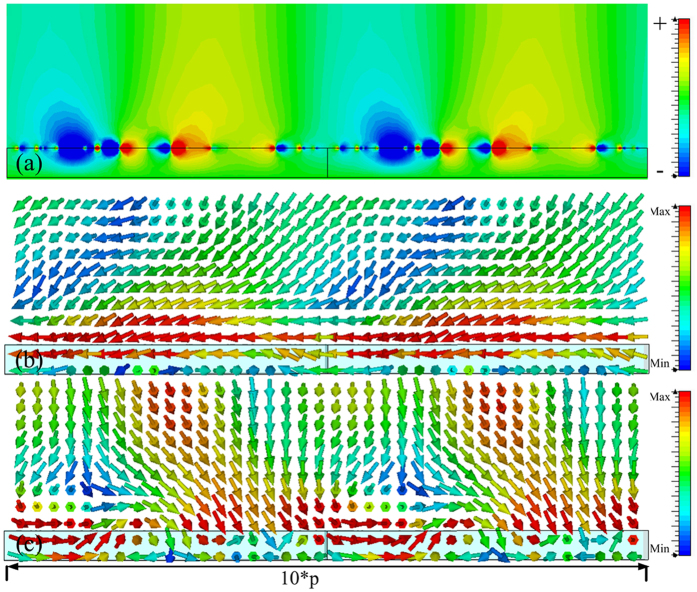
(**a**) The magnetic field in *z* direction of the spoof SPP at 7.574 GHz. (**b**) The power flow for left circularly polarized wave illumination at 7.574 GHz. (**c**) The power flow for right circularly polarized wave illumination at 7.574 GHz.

In order to verify the reduced reflection shown in Fig. 4, which was caused by the spoof SPPs coupling, the power flows in the *x-z* plane and the magnetic field were observed at 6.64 GHz, 6.835 GHz, 7.33 GHz and 7.574 GHz, and obtained results are presented in Figs 5, 6, 7 and 8, respectively. The presented results show that the *z* component of the magnetic field was generated on the metasurface at 6.64 GHz, 6.835 GHz, 7.33 GHz and 7.574 GHz. These generated field is evanescent in the *z* direction, which shows the localized field enhancement on the metasurface. The power flows at 6.64 GHz, 6.835 GHz, 7.33 GHz and 7.574 GHz for left and right circularly polarized wave illumination are also shown in Figs 5, 6, 7 and 8, respectively. The wave vector provided by the metasurface has opposite directions for left and right circularly polarized waves, as shown in Fig. 2(b) and (c). Consequently, the spoof SPPs coupling has opposite directions for left and right circularly polarized wave illumination. As shown in Figs 5, 6, 7 and 8, the proposed metasurface realizes helicity dependent directional spoof SPPs coupling.

**Figure 9:**
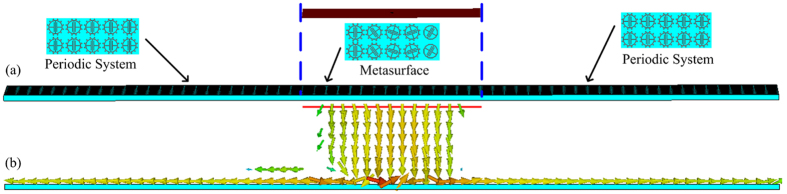
(**a**) Metasurface and periodic, (**b**) The power flow under partial linearly polarized wave illumination.

In order to further verify the spoof SPPs coupling, a periodic system is designed to extract coupling spoof SPPs on the proposed metasurface, and the power at 6.835 GHz is observed. The periodic system and the proposed metasurface^15^ are shown in Fig. 9(a). The periodic system is comprised of the unit cell, which has same geometric parameters with the unit 2 of the proposed gradient metasurface, Thus, the periodic system can also support the eigen spoof SPPs mode shown in Fig. 1(b). The periodic system is placed at both left and right side of the proposed metasurface, as shown in Fig. 9(a).

A linearly polarized wave can be decomposed into two circularly polarized waves, these two circularly polarized wave are right circularly polarized wave (RCP) and left circularly polarized wave (LCP). The proposed metasurface likes a Pancharatnam-Berry metasurface, the phase gradient of the proposed metasurface shown in Fig. 2(b) and (c) has opposite direction for right circularly polarized wave (RCP) and left circularly polarized wave^31^,^32^ (LCP). As shown in Fig. 9, a linearly polarized wave normally illuminates onto the middle part of whole system, which is the proposed metasurface. The decomposed RCP and LCP incidence are coupled into spoof SPPs along x-direction and -x-direction, respectively. Then, the coupled spoof SPPs is guided on the periodic system along x-direction and -x-direction simultaneously. Thus, helicity-controlled spoof SPPs coupling is also verified.

## Experiment results

**Figure 10:**
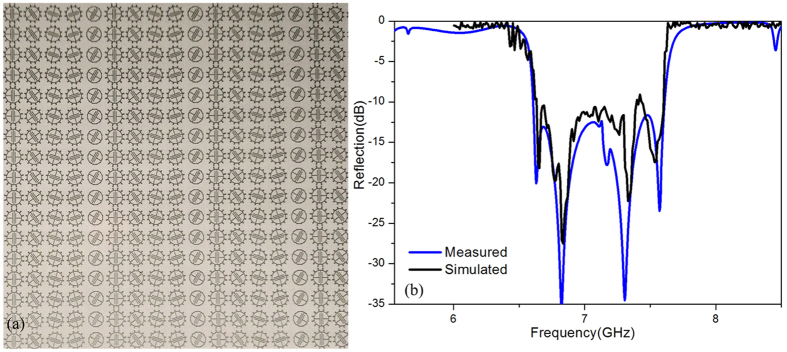
(**a**) The fabricated metasurface sample. (**b**) The measured reflection versus frequency for circularly polarized light.

Furthermore, the design of the proposed metasurface is verified by measurements; the large sample of the metasurface (410 mm × 410 mm), shown in Fig. 10(a), was fabricated. The fabricated metasurface sample has the same parameters as the one used in simulation of the Taconic RF-60A, whose dielectric constant and loss tangent are 6.15 and 0.0038, respectively. In order to measure the reflection of the metasurface, the Agilent vector network analyzer E8363B was used. A circularly polarized horn antenna was used to radiate the EM wave normally onto the metasurface sample, while another antenna was used to receive the reflected wave from the sample. On this way the reflection can be measured as a function of frequency. The measured results are presented in Fig. 10(b). The measured reflection dip has a wide band, which matches well with frequency bands obtained through the simulations. The measured reflection is more than −10 dB from 6.65 GHz to7.58 GHz bandwidth, which confirms the high efficiency of the spoof SPPs coupling.

## Conclusion

The high efficiency and wideband helicity dependent spoof SPPs coupling on the gradient metasurface is presented. It is shown that the coupling frequency of the spoof SPPs on the metasurface can be ascertained by the dispersion relation. The geometrical parameters of the metasurface cells are carefully designed. As the result, the multiple different dispersion relations are achieved using only one metasurface. For the constant wave vector provided by the metasurface, the spoof SPPs coupling can occur at multiple frequencies, which leads to the wideband spoof SPPs coupling. Both the simulation and experiment have shown that the wideband spoof SPPs coupling has been achieved.

Moreover, the wideband spoof SPPs coupling is comprised by the nearby frequency of the spoof SPPs coupling, because the proposed metasurface has multiple different dispersion relations. Furthermore, if the dispersion curve of the metasurface overlaps the wave vector line in a wide band, the spoof SPPs coupling will occur at the whole correspond frequency range.

## Additional Information

**How to cite this article**: Dong, G. *et al*. Wideband helicity dependent spoof surface plasmon polaritons coupling metasurface based on dispersion design. *Sci. Rep.* **6**, 38460; doi: 10.1038/srep38460 (2016).

**Publisher's note:** Springer Nature remains neutral with regard to jurisdictional claims in published maps and institutional affiliations.

## References

[b1] Barnes W. L., Dereux A. & Ebbesen T. W. Surface plasmon subwavelength optics. Nature 424, 824–830 (2003). 12917696 10.1038/nature01937

[b2] Pendry J. B., Martin-Moreno L. & Garcia-Vidal F. J. Mimicking surface plasmons with structured surfaces. Science 305, 847–848 (2004). 15247438 10.1126/science.1098999

[b3] Hibbins A. P., Evans B. R. & Sambles J. R. Experimental verification of designer surface plasmons. Science 308, 670–672 (2005). 15860621 10.1126/science.1109043

[b4] Shen X. & Cui T. J. Planar plasmonic metamaterial on a thin film with nearly zero thickness. Appl. Phys. Lett. 102, 211909 (2013).

[b5] Xu J. J., Zhang H. C., Zhang Q. & Cui T. J. Efficient conversion of surface-plasmon-like modes to spatial radiated modes. Appl. Phys. Lett. 106, 021102 (2015).

[b6] Ma H. F., Shen X., Cheng Q., Jiang W. X. & Cui T. J. Broadband and high-efficiency conversion from guided waves to spoof surface plasmon polaritons. Laser & Photonics Rev. 8, 146–151 (2014).

[b7] Liu X. Y., Feng Y. J., Zhu B., Zhao J. M. & Jiang T. Backward spoof surface wave in plasmonic metamaterial of ultrathin metallic structure. Sci. Rep. 6, 20448 (2016). 26842340 10.1038/srep20448 PMC4740866

[b8] Kats M. A., Woolf D., Blanchard R., Yu N. & Capasso F. Spoof plasmon analogue of metal-insulator-metal waveguides. Opt. Exp. 19, 14860–14870 (2011). 10.1364/OE.19.014860 21934847

[b9] Lan Y.-C. & Chern R.-L. Surface plasmon-like modes on structured perfectly conducting surfaces. Opt. Exp. 14, 11339– 11347 (2006). 10.1364/oe.14.011339 19529551

[b10] Hibbins A. P., Hendry E., Lockyear M. J. & Sambles J. R. Prism coupling to ‘designer’ surface plasmons. Opt. Exp. 16, 20441–20447 (2008). 10.1364/oe.16.020441 19065182

[b11] Chen Y.-H. . Direct observation of amplified spontaneous emission of surface plasmon polaritons at metal/dielectric interfaces. Appl. Phys. Lett. 98, 261912 (2011).

[b12] LeGall J., Olivier M. & Greffet J. J. Experimental and theoretical study of reflection and coherent thermal emission by a SiC grating supporting a surface-phonon polariton. Phys. Rev. B 55, 10105–10114 (1997).

[b13] Yu N. . Light Propagation with Phase Discontinuities: Generalized Laws of Reflection and Refraction. Science 334, 333–337 (2011). 21885733 10.1126/science.1210713

[b14] Sun S. . High-Efficiency Broadband Anomalous Reflection by Gradient Meta-Surfaces. Nano Lett. 12, 6223–6229 (2012). 23189928 10.1021/nl3032668

[b15] Sun S. . Gradient-index meta-surfaces as a bridge linking propagating waves and surface waves. Nat. Mater. 11, 426–431 (2012). 22466746 10.1038/nmat3292

[b16] Aieta F. . Out-of-Plane Reflection and Refraction of Light by Anisotropic Optical Antenna Metasurfaces with Phase Discontinuities. Nano Lett. 12, 1702 (2012). 22335616 10.1021/nl300204s

[b17] Yu N. & Capasso F. Flat optics with designer metasurfaces. Nat. Mate. 13, 139–150 (2014). 10.1038/nmat3839 24452357

[b18] Dong G. . Ultra-broadband perfect cross polarization conversion metasurface. Opt. Commun. 365, 108–112 (2016).

[b19] Li Y. B., Cai B. G., Cheng Q. & Cui T. J. Surface Fourier-transform lens using a metasurface. J. Phy. D: Appl. Phy. 48, 035107 (2015).

[b20] Wan X., Li Y. B., Cai B. G. & Cui T. J. Simultaneous controls of surface waves and propagating waves by metasurfaces. Appl. Phys. Lett. 105, 121603 (2014).

[b21] Cai B. G., Li Y. B., Jiang W. X., Cheng Q. & Cui T. J. Generation of spatial Bessel beams using holographic metasurface. Opt. Exp. 23, 7593–7601 (2015). 10.1364/OE.23.007593 25837097

[b22] Li Y. B., Cai B. G., Wan X. & Cui T. J. Diffraction-free surface waves by metasurfaces. Opt. Lett. 39, 5888–5891 (2014). 25361111 10.1364/OL.39.005888

[b23] Ni X., Emani N. K., Kildishev A. V., Boltasseva A. & Shalaev V. M. Broadband Light Bending with Plasmonic Nanoantennas. Science 335, 427–427 (2012). 22194414 10.1126/science.1214686

[b24] Li Y. . Wideband radar cross section reduction using two-dimensional phase gradient metasurfaces. Appl. Phys. Lett. 104, 221110 (2014).

[b25] Farmahini-Farahani M. & Mosallaei H. Birefringent reflectarray metasurface for beam engineering in infrared. Opt. Lett. 38, 462–464 (2013). 23455103 10.1364/OL.38.000462

[b26] Larouche S. & Smith D. R. Reconciliation of generalized refraction with diffraction theory. Opt. Lett. 37, 2391–2393 (2012). 22739918 10.1364/OL.37.002391

[b27] Sun W. J., He Q., Sun S. L. & Zhou L. High-efficiency surface plasmon meta-couplers: concept and microwave-regime realizations. Light-Sci. Appl. 5, e16003 (2016). 10.1038/lsa.2016.3 PMC6059849 30167110

[b28] Wu C., Cheng Y., Wang W., He B. & Gong R. Ultra-thin and polarization-independent phase gradient metasurface for high-efficiency spoof surface-plasmon-polariton coupling. Appl. Phys. Express. 8, 12201 (2015).

[b29] Wu C. J., Cheng Y. Z., Wang W. Y., He B. & Gong R. Z. A polarization independent phase gradient metasurface for spoof plasmon polaritons coupling. J. Opt. 18, 025101 (2016).

[b30] Wang J. . High-efficiency spoof plasmon polariton coupler mediated by gradient metasurfaces. Appl. Phys. Lett. 101, 201104 (2012).

[b31] Huang L. . Dispersionless Phase Discontinuities for Controlling Light Propagation. Nano Lett. 12, 5750–5755, (2012). 23062196 10.1021/nl303031j

[b32] Huang L. . Helicity dependent directional surface plasmon polariton excitation using a metasurface with interfacial phase discontinuity. Light-Sci. Appl. 2, e70 (2013).

